# Velvet Antler Ameliorates Cardiac Function by Restoring Sarcoplasmic Reticulum Ca^2+^-ATPase Activity in Rats With Heart Failure After Myocardial Infarction

**DOI:** 10.3389/fphar.2021.621194

**Published:** 2021-04-30

**Authors:** Haoyue Shi, Tianzi Zhao, Yanjun Li, Xiang Xiao, Jiayun Wu, Haojun Zhang, Jiajun Qiao, Li Huang, Lin Li

**Affiliations:** ^1^ Department of Cardiology, Beijing Hospital of Traditional Chinese Medicine, Capital Medical University, Beijing, China; ^2^ Cardiovascular Internal Medicine, Affiliated Hospital of Hebei University of Traditional Chinese Medicine, Shijiazhuang, China; ^3^ Cardiovascular Department, Rizhao Traditional Chinese Medicine Hospital, Rizhao, China; ^4^ Traditional Chinese and Western Medicine of Integrative Cardiology, China-Japan Friendship Hospital, Beijing, China; ^5^ Graduate School of Beijing University of Chinese Medicine, Beijing University of Chinese Medicine, Beijing, China

**Keywords:** heart failure, velvet antler, SERCA2a, PLB, PKA

## Abstract

**Objective:** Velvet antler (VA; cornu cervi pantotrichum), a well-known traditional Chinese medicine, has been shown to exert cardioprotective effects. The purpose of this study was to investigate the effect of VA on heart failure (HF) caused by ischemia-reperfusion, and explore its possible mechanism from the regulation of sarcoplasmic/endoplasmic reticulum Ca^2+^-ATPase 2 alpha (SERCA2a).

**Methods:** A rat model of HF was established by ligating the left anterior descending coronary artery of male Sprague–Dawley rats (*n* = 88). One week after surgery, VA (200, 400, or 800 mg/[kg day^−1^]) or enalapril (1 mg/[kg day^−1^]) was administered daily for the next 4 weeks. Heart function was detected by echocardiography and histopathological analysis. The serum BNP level was measured by ELISA, and the expression of SERCA2a, PLB, PLB-Ser^16^, and PKA was determined by western blotting. SERCA2a and PLB mRNA levels were determined by real-time quantitative PCR.

**Results:** Compared with the sham group, cardiac function in the HF group, including the serum BNP level, heart mass index, myocardial collagen deposition, and left ventricular ejection fraction, was markedly reduced; however, these changes could be reversed by VA treatment. In addition, VA (200 mg/[kg·d^−1^]) inhibited the decrease of SERCA2a and PLB mRNA levels and SERCA2a, PLB, PLB-Ser^16^, and PKA protein expression and restored the activity of SERCA2a and PKA. Enalapril affected only PLB protein expression.

**Conclusion:** VA can improve myocardial fibrosis and ventricular remodeling in rats, thereby helping to restore cardiac function. The underlying mechanism may be related to the upregulation of the expression and activation of PKA and PLB and the restoration of the expression and activity of SERCA2a.

## Introduction

Heart failure (HF), which affects between 1 and 2% of the world’s adult population (Tanai and Frantz, 2015), is a complex clinical syndrome caused by ventricular filling or the ejection of blood (Yancy et al., 2013). The last 20 years have seen substantial progress in HF therapy, and the treatment strategy has changed from cardiac strengthening, diuresis, and vasodilation to inhibition of the neuroendocrine system and ventricular reconstruction. Even though survival has improved, HF remains a major healthcare problem, with an estimated prevalence of more than 26 million cases worldwide (Ponikowski et al., 2014; Rajadurai et al., 2017). Approximately 4 million people in China are estimated to have HF (Guo et al., 2018), while in the United States the figure is nearly 5.1 million (Go et al., 2013). This highlights the urgent need to identify new and effective approaches for the treatment of HF.

The activity of sarcoplasmic/endoplasmic reticulum Ca^2+^-ATPase 2 alpha (SERCA2a) tightly regulates cardiac contractility by performing active reuptake of cytoplasmic Ca^2+^. Whereas impaired SERCA2a function is associated with HF, restoring the function of SERCA2a represents a therapeutic strategy to recover cardiac performance (Bers, 2002; Sitsel et al., 2019). SERCA2a controls cardiac muscle relaxation by regulating the amount of Ca^2+^ released during myocardial contraction, which, in turn, determines the extent of cardiac muscle contraction (Bers, 2002). Several transmembrane micropeptides regulate the activity of SERCA2a in the heart, including phospholamban (PLB) (MacLennan and Kranias, 2003), cAMP-dependent protein kinase (PKA), and dwarf open reading frame (DWORF) (Nelson et al., 2016). If SERCA2a expression and PLB phosphorylation are reduced, Ca^2+^ uptake into the sarcoplasmic reticulum (SR) will be negatively affected, thereby impairing the contractile performance of the heart (Sitsel et al., 2019). Consequently, a reduction in SERCA2a activity can lead to the progressive deterioration of cardiac contractility in patients with HF.

Velvet antler (VA; cornu cervi pantotrichum), a traditional Chinese medicine mentioned in the “Compendium of Materia Medica”, is one of the few mammalian organs that can continuously regenerate. VA is not only a precious traditional Chinese medicine, but is also widely used in other countries, for example, it has become a part of Russian officinal medicine as an animal-derived medicinal product (ADMP) since 1778 (Prokopov et al., 2019). It has been reported that VA extract can promote chondrocyte proliferation and enhance sexual performance in old male mice (Zang et al., 2015). We have previously shown that VA can improve heart function, ameliorate ischemia–hypoxia-induced cardiac microvascular endothelial cell injury, and repair vascular endothelial cell damage (Shao et al., 2012; Xiang et al., 2017; Li et al., 2018). Based on these observations, we further explored the mechanism by which VA improves cardiac function in rats with HF after myocardial infarction, and found that it likely involves the restoration of SERCA2a activity.

## Methods

### Drug Preparation

Velvet antlers in the early stages of growth and development were obtained from a 4 year-old sika deer (*Cervus nippon*) provided by the Qingyuan Manchu Autonomous County of Science and Technology Development Center in Changchun, Jilin Province, China. The deer was first placed under general anesthesia and the antler was then removed proximally using a surgical hand saw. The antlers were then cleaned with 75% ethanol and the blood was extracted using a vacuum instrument (SHZIIIB, Tan Shi Vacuum Equipment Co., Ltd., Linhai, Zhejiang, China). After freeze-drying, the VA was sliced and ultrafine crushing technology was used to prepare the VA powder under low temperature. The dehydration percentage of VA was 67.02%. The VAs were identified as young, unossified antlers from *Cervus nippon* by professor Chunsheng Liu from the Department of Chinese Medicine Identification at the Institute of Traditional Chinese Medicine, Beijing University of Chinese Medicine. The VA was stored at −80°C. The VA powder was mixed with 0.5% carboxymethyl cellulose sodium salt (CMC-Na) for gavage. The conventional clinical dose of VA is 4 g/d (about 70 kg of body weight for adults). The human equivalent dose is calculated according to the animal dose conversion table and the body surface area (Sevilla et al., 2004). If the dose of rats is seven times lower than that of humans, then the equivalent dose of rats is 400 mg/kg/d, which we set as the middle-dose group. And we set the low and high dose groups at half and two times the concentration, respectively. At the same time, enalapril was used as a positive control as it belongs to the angiotensin-converting enzyme (ACE) inhibitor drug class—cornerstone drugs for the treatment of HF (Consensus Trial Study Group, 1987)—and also because studies have shown that enalapril can alter the contractile performance and arrhythmogenicity of healthy myocardium in rats by upregulating SERCA2a (Matus et al., 2015). According to the instructions for enalapril, the conventional dose of enalapril is 10 mg/d (about 70 kg of body weight for adults), so the equivalent dose for rats is 1 mg/kg/d. Enalapril maleate (H32026567) was provided by the Yangtze River Pharmaceutical Group Jiangsu Pharmaceutical Co., Ltd. Primary antibodies against SERCA2a, PLB, PLB-Ser16, and PKA were obtained from Abcam (Cambridge, MA, United States). The BNP ELISA Kit was provided by the Beijing Huanya Biomedicine Technology Co., Ltd. (Beijing, China). The SERCA2a activity detection kit was obtained from the Nanjing Jiancheng Biology Engineering Institute (Nanjing, China), and the PKA activity kit from the Promega Biotechnology Co., Ltd. (Beijing, China).

### Liquid Chromatography–Mass Spectrometry Analysis and Mass Spectrometry Data Analysis

The VA used here was from the same batch of antlers as that used in our previous study (Xiang et al., 2017). Protein is the most important bioactive component in VA (Yu et al., 2017), so we mainly analyzed the protein of VA (VA-Pro). For LC, the liquid was separated with a C18 column (2.1 × 100 mm, 3 μm; Thermo Hypersil Gold) at a constant flow rate of 0.35 ml/min. The MS data were acquired using Xcalibur 4.1 (Thermo Fisher Scientific, Waltham, MA, United States), and the metabolites were identified by OSI/SMMS (Dalian ChemDataSolution Information Technology Co., Ltd., China). LC–MS analysis was performed as previously described (Xiang et al., 2017). After detection by LC-MS, we imported the original data into the PD (Proteome Discoverer 1.3, Thermo Fisher Scientific, Waltham, MA, United States), and processed the data using mascot server (version 2.3.0, Matrix Science) and the UniProt database. Annotated based on the GO database and KEGG database, VA-Pro has a total of 386 proteins identified by the MASCOT search. In the further enrichment analysis, we found that VA-Pro was mainly involved in the process of apoptosis in the biological process. In the metabolic process, VA-Pro was mainly involved in the biological process of metabolic regulation and response to stimulation. In addition, it was involved in the process of cell metabolism and regulation of cell metabolism (Xiang et al., 2017) (Supplementary Table S1).

### Animals

Eight-week-old male Sprague–Dawley (SD) rats weighing 210–230 g were obtained from the Beijing Hua-Fu-Kang Biological Technology Co., Ltd. (Certificate no. SCXK (jing) 2009–0007). The animals were kept at a temperature of 21–23°C, humidity of 40–60%, and a 12 h light/dark cycle with *ad libitum* access to water and chow diet. All experimental procedures were approved by the Institutional Animal Care and Use Committee (IACUC) and were performed in accordance with the Guiding Principles for the Care and Use of Laboratory Animals of the Institute of Materia Medica, Chinese Academy of Medical Science and Peking Union Medical College. A total of 45 rats were randomly divided into two groups, namely, a sham group (*n* = 6) and a surgery group (*n* = 39). The rats were anesthetized with sodium pentobarbital (50 mg/kg, i.p.). To maintain breathing, the rats were connected to a positive-pressure respirator (ALC-V8, Shanghai, China) *via* an intratracheal cannula. The chest was opened between the third and fourth ribs on the left-hand side by thoracotomy. The heart was quickly exposed and the left anterior descending coronary artery was ligated between the left atrium and pulmonary artery cone (1–1.5 mm from the left atrium). Heart rate was monitored on a 12-lead electrocardiograph (fx7203, Fukuda, Japan), and J-point elevations ≥ 2 indicated that the model was successful. Rats in the sham group underwent the surgical procedure but without ligation. After recovering from anesthesia, the animals were housed separately for one day and were allowed water and rodent chow. Six rats died of HF or arrhythmia after surgery. 1 week after the surgery, the rats were again anesthetized with sodium pentobarbital (30 mg/kg, i.p.) and three rats were examined by electrocardiography. Rats with elevated Q waves in leads V2 to V6 were considered to be HF models. The rats were then randomly divided into the following five groups: a HF group (*n* = 6); a HF + low-dose VA group (200 mg/[kg d^−1^]) (*n* = 6); a HF + medium-dose VA group (400 mg/[kg d^−1^]) (*n* = 6); a HF + high-dose VA group (800 mg/[kg d^−1^]) (*n* = 6); and a HF + enalapril group (1 mg/[kg d^−1^]) (*n* = 6). 4 weeks after drug intervention, the rats underwent echocardiography and were subsequently sacrificed.

### Echocardiography

Left ventricle function was evaluated using a Vevo 770 High-Resolution Imaging System (Visual Sonics Inc., Toronto, ON, Canada) with a 17.5 MHz linear array transducer. Left ventricular ejection fractions (LVEFs) and left ventricular fractional shortening (LVFS) were obtained from a long-axis view of the left ventricle, and the values were the average of five cardiac cycles.

### Heart Mass Index

The hearts were collected after being perfused with saline. Both body weight (BW) and heart weight (HW) were obtained, and the HW: BW ratio represented the HMI.

### Histological Examination

The hearts were fixed in 4% paraformaldehyde for 24 h, paraffin-embedded, and sliced into 5 μm sections. The sections underwent hematoxylin and eosin (H&E) and Masson’s trichrome staining to observe myocardial hypertrophy and collagen deposition, respectively. Six fields of each section were randomly selected for measuring the survival myocardium area (SMA) and the collagen area (CA) under an optical microscope. The images were analyzed using Image-Pro Plus 6.0 software (Media Cybernetics, Bethesda, MD, United States); the fibrotic area ratio (FAR) was calculated as FAR = CA/(CA + SMA) × 100%.

### Enzyme-Linked Immunosorbent Assay

Blood was collected from the abdominal aorta of the rats, and collected in the EDTA test tube, centrifuged at 2000 r/min, and the supernatant was collected and stored in the refrigerator at −20°C. The contents of BNP were determined using the ELISA kit (Abcam, ab108816, United Kingdom) according to the manufacturer’s instructions.

### Western Blotting Analysis

Myocardial tissue was obtained from each animal and snap-frozen in liquid nitrogen. Whole protein was extracted using a protein extraction kit, separated by sodium dodecyl sulfate–polyacrylamide gel electrophoresis (SDS–PAGE), and transferred to nitrocellulose membranes. The membranes were incubated overnight at 4°C with antibodies against SERCA2a, PLB, PLB-Ser16, or PKA and then with secondary antibody for 1 h at room temperature. Immunoreactive bands were revealed using enhanced chemiluminescence.

### Real-Time Quantitative Polymerase Chain Reaction

Real-time qPCR was performed to detect the mRNA levels of SERCA2a and PLB in the myocardial tissue of each mouse. Briefly, total RNA was extracted using an RNeasy Fibrous Tissue Mini Kit (Qiagen, Hilden, Germany). qPCR was performed in an ABI PRISM 7500 Sequence Detection System (Perkin–Elmer Applied Biosystems, CA, United States). The following primers were used: Serca2a-forward, 5′-CAC​CTG​GAA​GAT​TCT​GCG​AAC​T-3′ and Serca2a-reverse, 5′-CAT​CCT​CAT​CCT​GCC​CAA​AG-3′; PLB-forward, 5′-GGA​GCC​TGT​GTC​CTT​GTG​TC-3′ and PLB-reverse, 5′-GAA​GTC​ATC​CCT​GGT​GTC​GT-3′; ACTIN-forward, 5′- GGA​GAT​TAC​TGC​CCT​GGC​TCC​TA-3′ and ACTIN-reverse, 5′-GAC​TCA​TCG​TAC​TCC​TGC​TTG​CTG-3′. All reactions were performed in triplicate.

### Statistical Analysis

All parameters were expressed as means ± SEM. Statistical analysis was performed in SPSS 19.0. The data were analyzed by one-way analysis of variance followed by Tukey’s test for multiple comparisons. A *p* value < 0.05 was considered statistically significant.

## Results

### The Effects of Velvet Antler on Cardiac Function

**FIGURE 1:**
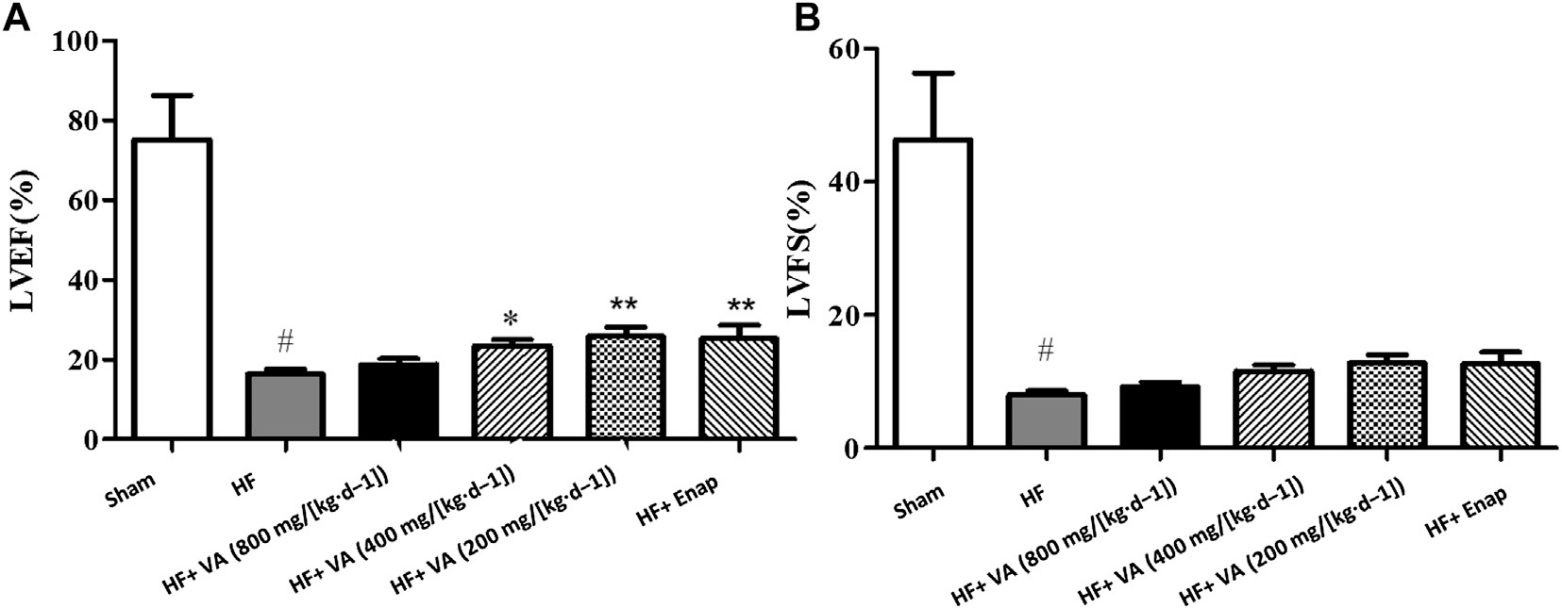
The effects of velvet antlers (VA) on the left ventricular ejection fraction (LVEF) **(A)** and left ventricular fractional shortening (LVFS) **(B)**. The data are expressed as means ± SEM. #: *p* < 0.05 vs. the sham group; *: *p* < 0.05 vs. the heart failure (HF) group; **: *p* < 0.01 vs. the HF group; *n* = 6. Enap: enalapril.

Compared with the sham group, the hearts of rats in the HF group exhibited marked cardiac dysfunction as evidenced by a significant reduction of LVEFs and LVFS (Figures 1A,B). Treatment with VA at low (200 mg/[kg·d^−1^]) and medium 400 mg/[kg·d^−1^] doses, but not at a high dose (800 mg/[kg·d^−1^]), significantly improved LVEFs after HF (Figure 1A). However, neither VA nor enalapril had a significant effect on LVFS (Figure 1B).

### The Effect of Velvet Antler on the Heart Mass Index

**FIGURE 2:**
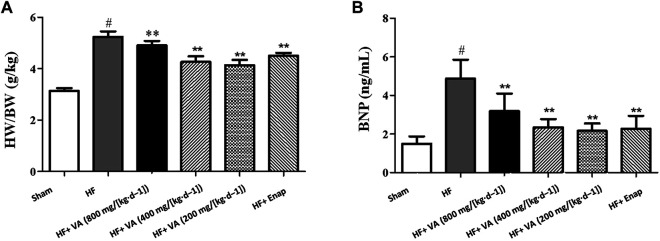
The effect of velvet antlers (VA) on the heart mass index (HMI) **(A)** and serum BNP levels **(B)**. The data are expressed as means ± SEM. #: *p* < 0.05 vs. the sham group; *: *p* < 0.05 vs. the heart failure (HF) group; **: *p* < 0.01 vs. the HF group. *n* = 6. HW: heart weight; BW: body weight; Enap: enalapril.

The HMI was markedly higher in the HF group than in the sham group. Treatment with all three VA doses or with enalapril significantly reduced the HMI when compared with that of the model group (Figure 2A).

### The Effect of Velvet Antler on Serum Levels of BNP

The serum level of BNP was greatly increased in the HF group when compared with that in the sham group. Treatment with all three VA doses significantly reduced BNP levels after HF, and the same was observed for enalapril (Figure 2B).

### The Effect of Velvet Antler on Morphology and Collagen Deposition in Myocardial Tissue

**FIGURE 3:**
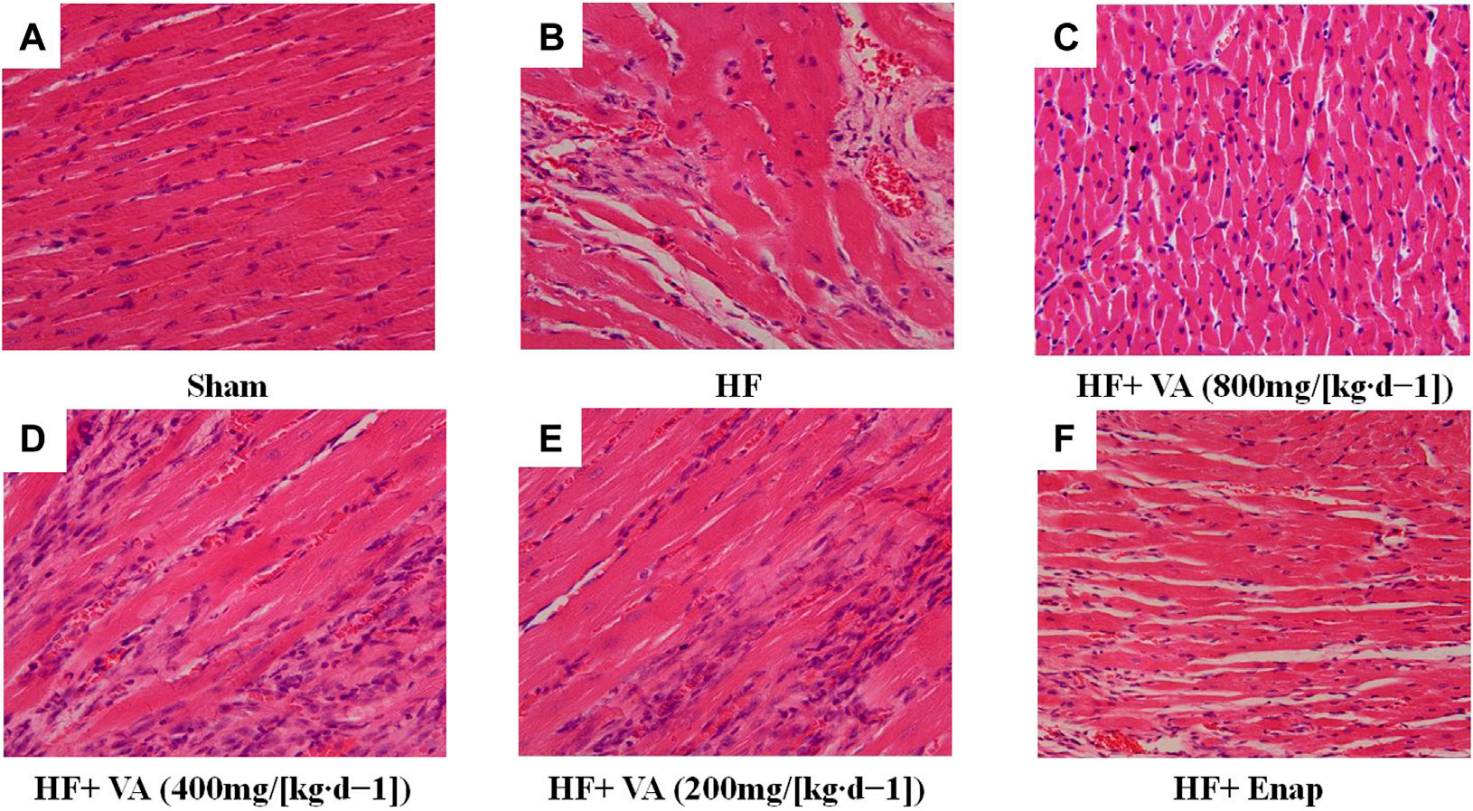
The effect of velvet antlers (VA) on myocardial histology. **(A**–**F)** Representative myocardial sections of the sham group **(A)**, heart failure (HF) group **(B)**, HF + low dose of VA group **(C)**, HF + medium dose of VA group **(D)**, HF + high dose of VA group **(E)**, and HF + Enap (enalapril) group **(F)** stained with hematoxylin and eosin (H&E). Bar = 50 µm.

The results of the histological examinations for each group are presented in Figure 3. In the sham group, myocardial fiber was regularly arranged, with the nucleus distributed in the center of the muscle cells (Figure 3A). In contrast, distinct alterations could be seen in the HF group, including myocardial necrosis, myocardial fiber disruption, interstitial edema, and inflammatory cell infiltration (Figure 3B). The HF-induced morphological changes in myocardial tissue were ameliorated by treatment with both VA and enalapril (Figures 3C–F).

**FIGURE 4:**
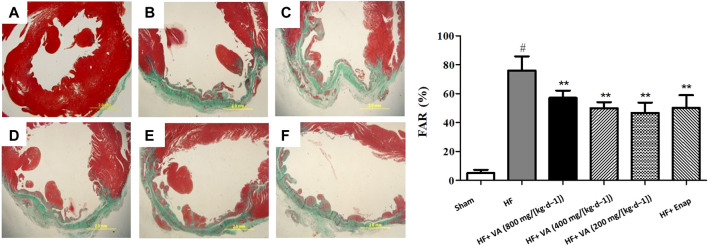
The effect of velvet antlers (VA) on myocardial collagen deposition. **(A)** Representative myocardial tissue of the sham group **(a)**, heart failure (HF) group **(b)**, HF + low dose of VA group **(c)**, HF + medium dose of VA group **(d)**, HF + high dose of VA group **(e)**, and HF + enalapril group **(f)** stained with Masson’s trichrome. Bar = 20 mm. **(B)** Statistical analysis of the fibrotic area ratio (FAR) in each group. The data are expressed as means ± SEM. #: *p* < 0.01 vs. the sham group; *: *p* < 0.05 vs. the HF group; **: *p* < 0.01 vs. the HF group. *n* = 6.

Noticeable collagen deposition was observed in the myocardium of rats from the HF group, as demonstrated by Masson’s trichrome staining (Figure 4A, green) and an increase in the FAR (Figure 3B). Treatment with all three VA doses or with enalapril significantly increased collagen fiber hyperplasia after HF (Figures 3B, 4A).

### The Effect of Velvet Antler on Sarcoplasmic/Endoplasmic Reticulum Ca^2+^-ATPase 2 alpha and Phospholamban mRNA Levels

**FIGURE 5:**
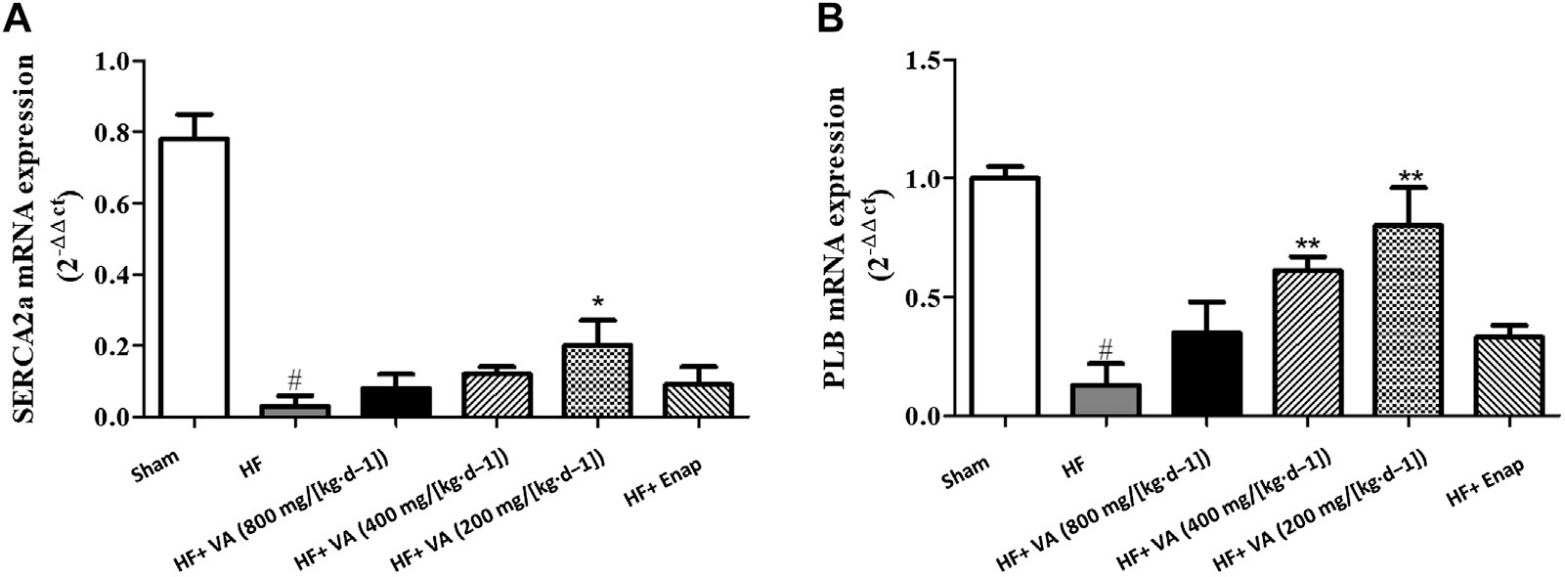
The effect of velvet antlers (VA) on SERCA2a **(A)** and PLB **(B)** mRNA expression. The data are expressed as means ± SEM. #: *p* < 0.01 vs. the sham group; *: *p* < 0.05 vs. the heart failure (HF) group; **: *p* < 0.01 vs. the HF group. *n* = 6. Enap: enalapril.

Compared with those of the sham group, the mRNA levels of SERCA2a were markedly decreased in the HF group; however, this effect could be inhibited by treatment with a low dose 200 mg/[kg·d^−1^] of VA (Figure 5A). The PLB mRNA level was significantly reduced after HF, but this reduction could be rescued by treatment with low 200 mg/[kg·d^−1^] or medium 400 mg/[kg·d^−1^] doses of VA (Figure 5B). However, neither the high 800 mg/[kg·d^−1^] VA dose nor enalapril increased the mRNA levels of SERCA2a or PLB (Figure 5).

### The Effect of Velvet Antler on the Protein Expression of Sarcoplasmic/Endoplasmic Reticulum Ca^2+^-ATPase 2 alpha, Phospholamban, and Protein Kinase

**FIGURE 6:**
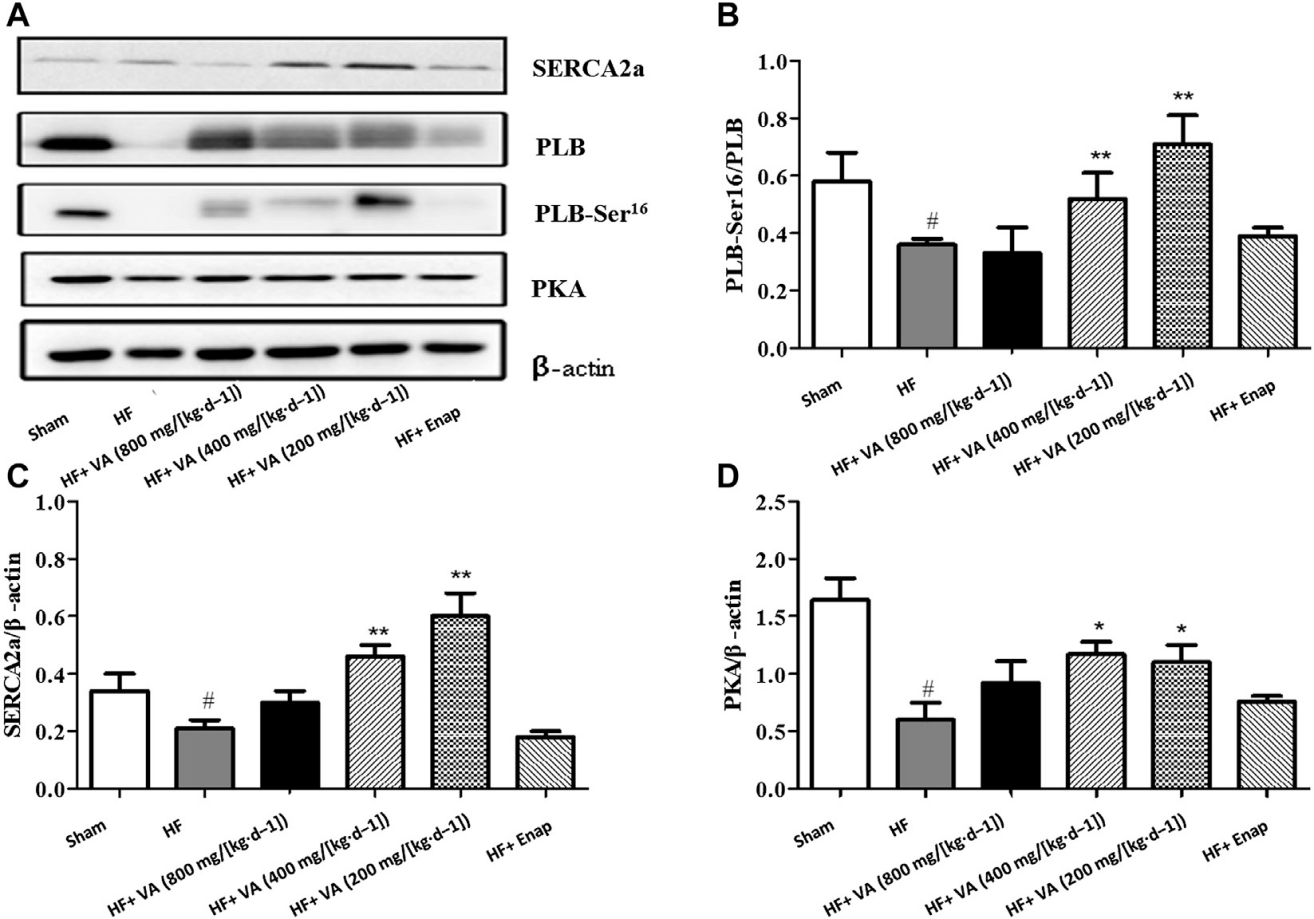
The effect of velvet antlers (VA) on the protein expression of SERCA2a, PLB, PLB-Ser^16^, and PKA. **(A)** Representative western blot of each protein in the different groups. **(B–D)** Quantitative analysis of SERCA2a **(B)**, phosphorylated PLB **(C)**, and PKA **(D)** expression. The data are expressed as means ± SEM. #: *p* < 0.01 vs. the sham group; *: *p* < 0.05 vs. the heart failure (HF) group; **: *p* < 0.01 vs. the HF group. *n* = 6. Enap: enalapril.

The protein expression of SERCA2a, as well as that of its downstream effectors PLB and PKA, was significantly lower in the HF group than in the sham group; however, these effects could be inhibited by low 200 mg/[kg·d^−1^] and medium 400 mg/[kg·d^−1^] doses of VA (Figure 6). Furthermore, PLB phosphorylation on serine 16 was increased by VA treatment (Figure 6). Enalapril did not affect the levels of the above-mentioned proteins (Figure 6).

### The Effect of Velvet Antler on the Activity of Sarcoplasmic/Endoplasmic Reticulum Ca^2+^-ATPase 2 alpha and Protein Kinase

**FIGURE 7:**
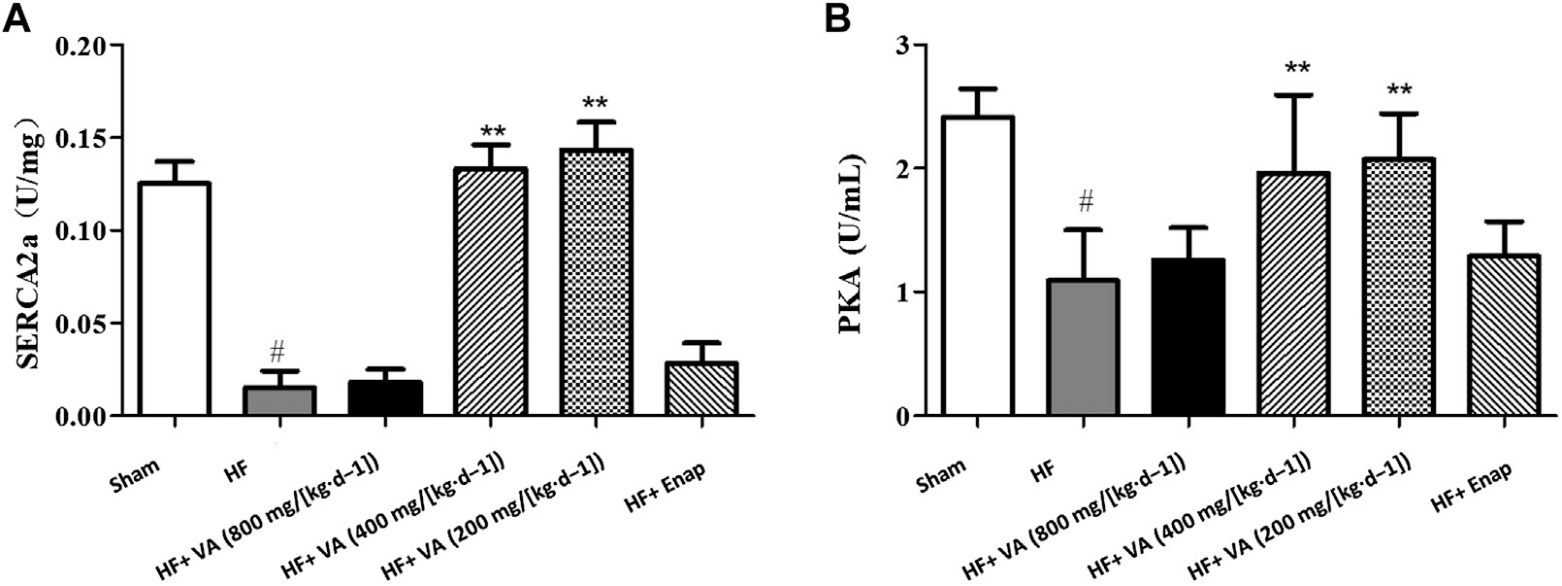
The effect of velvet antlers (VA) on SERCA2a **(A)** and PKA **(B)** activity. The data are expressed as means ± SEM. #: *p* < 0.01 vs. the sham group; *: *p* < 0.05 vs. the heart failure (HF) group; **: *p* < 0.01 vs. the HF group. *n* = 6. Enap: enalapril.

Compared with the sham group, the activity of SERCA2a and its downstream effector protein PKA was significantly decreased in the HF group. These effects could be rescued by treatment with low 200 mg/[kg·d^−1^] and medium 400 mg/[kg·d^−1^] doses of VA (Figures 7A,B). However, neither the high 800 mg/[kg·d^−1^] dose of VA nor enalapril affected SERCA2a and PKA activity (Figure 7).

## Discussion

HF is the end stage of most heart diseases. Although treatments for HF can be effective, HF remains a leading cause of hospitalization globally (Jencks et al., 2009; Mozaffarian et al., 2015). Additionally, HF is associated with high health care costs, likely because the efficacy of most drugs currently used for the long-term prevention of HF in high-risk patients is limited by their side effects (Moe et al., 2015). Accordingly, it is important that new types of cardioprotective drugs are identified. In many cases of cardiac disease, altered Ca^2+^ cycling precedes the observed reduction in mechanical performance, suggesting that improving Ca^2+^ cycling could be an effective therapeutic strategy for HF prevention or treatment (Gorski et al., 2015). SERCA2a is a key regulator of Ca^2+^ concentrations during the cardiac cycle, controlling the transport of Ca^2+^ to the SR during cardiac muscle relaxation (Gianni et al., 2005). PLB, a single-span membrane protein of only 52 amino acids, can modulate SERCA2a activity in cardiac muscle. In its dephosphorylated form, PLB binds SERCA2a and actively inhibits Ca^2+^ transport (Tada et al., 1979), and phosphorylation of PLB reverses this inhibition. Serine 16, which is phosphorylated by PKA, is one of the distinct phosphorylation sites of PLB (Fujii et al., 1991; Moon et al., 2012).

VA has been a valuable herb in China since ancient times. According to the “Compendium of Materia Medica”*,* VA is the undifferentiated horn of young deer, which grows at a rate of 1–2 cm per day. It takes only 90 days to mature from the initiation of bone cell differentiation, and was used in the past for invigorating the kidney and nourishing the blood. In the present study, we showed that treatment with a low (200 mg/[kg d^−1^]) or medium (400 mg/[kg d^−1^]) dose of VA, which is equivalent to the clinical dosage (2–4 g/day), exerted positive effects on cardiac function in rats with HF after myocardial infarction. Further mechanistic analysis demonstrated that the mRNA and protein expression levels of SERCA2a, as well as its activity, were markedly decreased after HF. This is consistent with the results of previous study (Zarain-Herzberg et al., 2012) that suggested that decreased SERCA2a activity may have a direct relationship with cardiac dysfunction and cardiomyocyte enlargement (Fernandes et al., 2015). Consequently, SERCA2a is now regarded as a novel gene target for HF therapy in the clinic (Bouyon et al., 2014; Chen et al., 2019). Surprisingly, VA treatment could significantly restore SERCA2a mRNA and protein expression levels, thereby also restoring SERCA2a activity and expression. Because these results suggested that the ameliorative effect of VA on cardiac dysfunction may be closely related to the restoration of SERCA2a activity after HF, we further explored the underlying mechanisms. In the present study, the expression and phosphorylation of PLB declined after HF; however, the administration of VA reversed these changes. In addition to PLB expression and phosphorylation, we also investigated PKA activity, as PKA functions in the typical G protein-adenylate cyclase-cAMP-PKA signaling pathway and is key for PLB serine 16 phosphorylation (Nagatomo et al., 2009). Our results showed that PKA expression and activity were decreased in the HF group, but these effects could be reversed by VA treatment. The above results demonstrated that VA may enhance PLB phosphorylation through the PKA/PLB signaling pathway and inhibit the decrease of SERCA2a activity. In addition, the mRNA expression level of SERCA2a was significantly enhanced by VA treatment. PLB mainly affects the activity of SERCA2a without changing the expression of it, so we consider that there are other mechanisms that may upregulate SERCA2a expression, which still needs further research. Combined with clinical and previous studies, VA has the effect of stimulating androgen secretion (Zang et al., 2016). Androgen has been shown to improve cardiac function, which may be related to altering the key proteins involved in Ca^2+^ handling (Ribeiro et al., 2018; Barske et al., 2019). Besides, we found that there was no significant positive correlation between the dose and efficacy of VA, which was an interesting result. We reviewed the “Shennong Herbal sutra” and consulted the literature. VA may affect cardiac function through multiple pathways, and its concentration-effect relationship may be similar to that of dopamine, with its main mechanisms varying at different concentrations. The results of this study remind us that we may measure the blood pressure, heart rate, and urine volume of rats, and further subdivide them into different concentration groups to further measure hormones in order to elucidate the dose-efficacy relationship of VA. And from a therapeutic perspective, the effects at 200 mg/[kg d^−1^] are of therapeutic relevance. However, further research will be need to assess whether these effects can also be found at levels which are closer to a therapeutic dose. The results of this study provide a good idea for our further research.

In conclusion, we showed that VA treatment could ameliorate myocardial fibrosis and ventricular reconstruction, thereby improving cardiac function. The underlying mechanism may be related to the upregulation of the expression and activation of PKA and PLB and the restoration of SERCA2a expression and activity. These results provide evidence that VA has therapeutic potential for the treatment of coronary heart disease and HF.

## Data Availability

The original contributions presented in the study are included in the Supplementary Material, further inquiries can be directed to the corresponding author.
