# Supplementary material for: Velvet Antler Ameliorates Cardiac Function by Restoring Sarcoplasmic Reticulum Ca2+-ATPase Activity in Rats With Heart Failure After Myocardial Infarction
Source: Front Pharmacol. 2021 Apr 30;12:621194. doi: 10.3389/fphar.2021.621194 (PMC8120434; doi:10.3389/fphar.2021.621194)
Supplement: Supplementary file 1 [file datasheet1.docx]

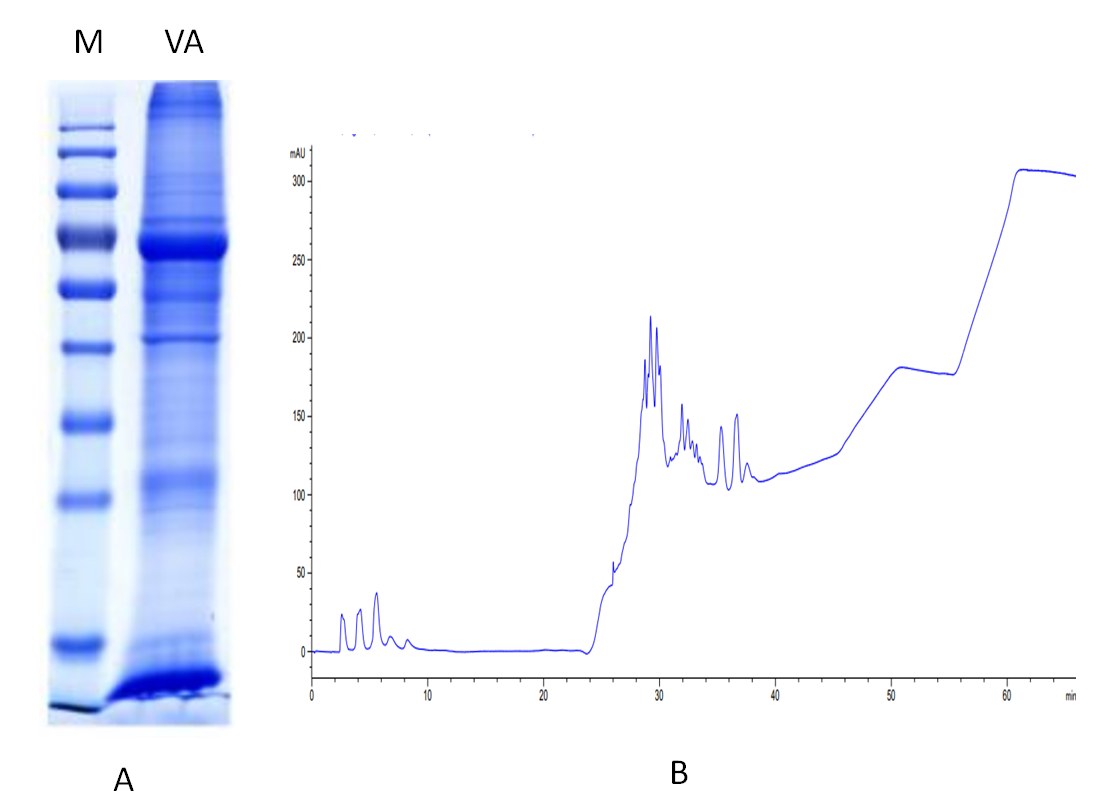


**
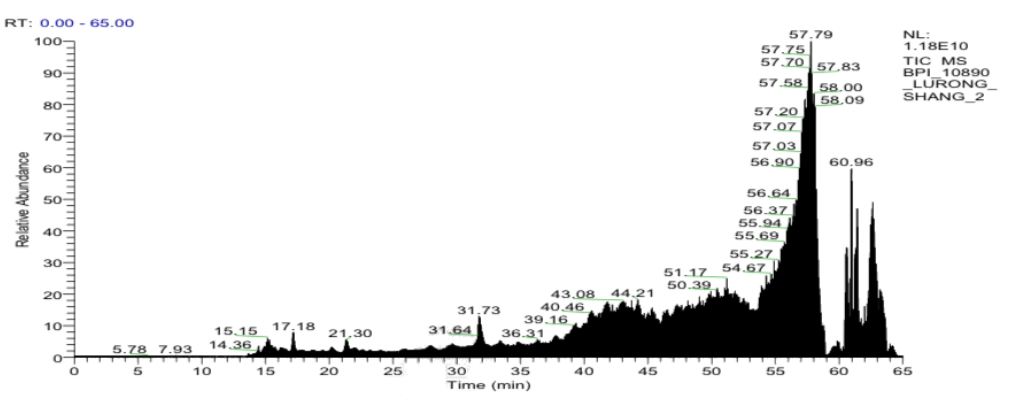
**

**C**

**
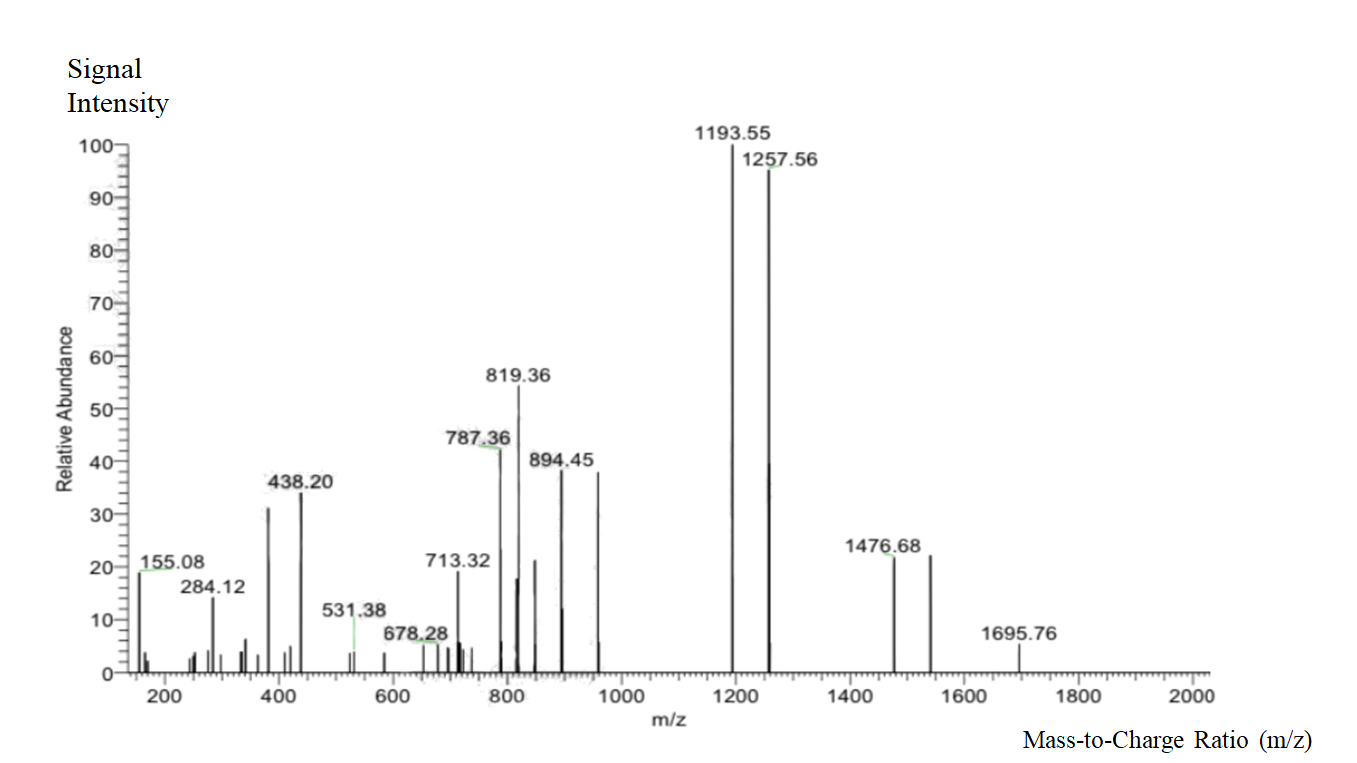
**

**D**

**A** Sds-page gluemap of VA macromolecular protein

**B** SCX separation figure of VA macromolecular protein

C Q-Exactive Mass Spectrum

D Secondary Mass Spectrum
